# Clinicopathological features and prognosis of malignant peripheral nerve sheath tumor: a retrospective study of 159 cases from 1999 to 2016

**DOI:** 10.18632/oncotarget.18975

**Published:** 2017-07-04

**Authors:** Zhennan Yuan, Libin Xu, Zhenguo Zhao, Songfeng Xu, Xinxin Zhang, Ting Liu, Shuguang Zhang, Shengji Yu

**Affiliations:** ^1^ Department of Orthopaedics, National Cancer Center/Cancer Hospital, Chinese Academy of Medical Sciences and Peking Union Medical College, Beijing 100021, China

**Keywords:** malignant peripheral nerve sheath tumor, clinicopathological features, prognosis, Ki67, S-100

## Abstract

**Objective:**

To investigate the clinicopathological features and prognosis of malignant peripheral nerve sheath tumors (MPNST).

**Results:**

A total of 159 patients with MPNST were enrolled in the study. The ratio of male to female was 1.04 to 1. The median age was 40 (range: 5–76) years at the time of diagnosis. The 3- and 5-year overall survival rates were 50.0% and 43.0%, respectively. The median follow-up period was 31.0 (range: 2.0–199.0) months. Multivariate analysis showed that AJCC stage and S-100 were independent factors affecting overall survival (*p* < 0.05 for both). 3- and 5-year tumor-free survival rates for 140 completely resected patients were 40.0% and 34.0%, respectively. Multivariate analysis showed that AJCC stage, S-100 and Ki67 staining were independent factors of tumor-free survival (*p* < 0.05 for all).

**Materials and Methods:**

The clinical data of MPNST patients who were treated at Cancer Institute and Hospital, Chinese Academy of Medical Science from January 1999 to January 2016 was retrospectively reviewed.

**Conclusions:**

MPSNT is a highly aggressive tumor with poor prognosis and this study may be useful for prognostic assessment and management decisions. This had been largest documented retrospective study of MPSNT among Chinese populations. Some characteristics were different from those of foreign populations which may suggest the specificity of Chinese patients.

## INTRODUCTION

Malignant peripheral nerve sheath tumors (MPNSTs) were designated by the World Health Organization in 2002 to replace previous terminologies of “malignant schwannoma”, “malignant neurilemmoma”, “neurogenic sarcoma”, and “neurofibrosarcoma” [1]. MPNST is a rare disease accounting for 6% of soft tissue sarcomas [2]. They occur in three different contexts: sporadic in around 40% of all cases, associated with neurofibromatosis type 1 (NF1), the most frequent autosomal dominant genetic disorder, in 50% of cases [3–7], and as a consequence of previous radiation therapy (RT-induced) in around 10% [5–7]. Patients with NF1 have an estimated 8–12% lifetime risk of developing MPNST, mainly derived from a pre-existing plexiform neurofibroma [8]. MPNST behaves aggressively, with a high rate of local recurrence and a significant propensity to metastasize. Surgical resection represents the mainstay of therapy. The benefit of radiation and systemic chemotherapy, when commonly administered, is undetermined. Despite aggressive combined modality therapy, survival is dismal with 5-year survival rate of 35%–50% [3, 4, 9].

Recently, research data have shown that NF1, surgical margin status, and tumor size are significant predictors of survival in patients with MPNST. Molecular predictors such as TP53 and S-100 are also suggested [4, 10–12]. However, large cohort studies on Chinese MPNST patients are totally absent [11, 12]. Noteworthily, it had been the largest documented retrospective study of MPNST among Chinese populations up to now. Clinical and pathological prognostic predictors affecting local and/or distant recurrence were analyzed.

## RESULTS

**Figure 1:**
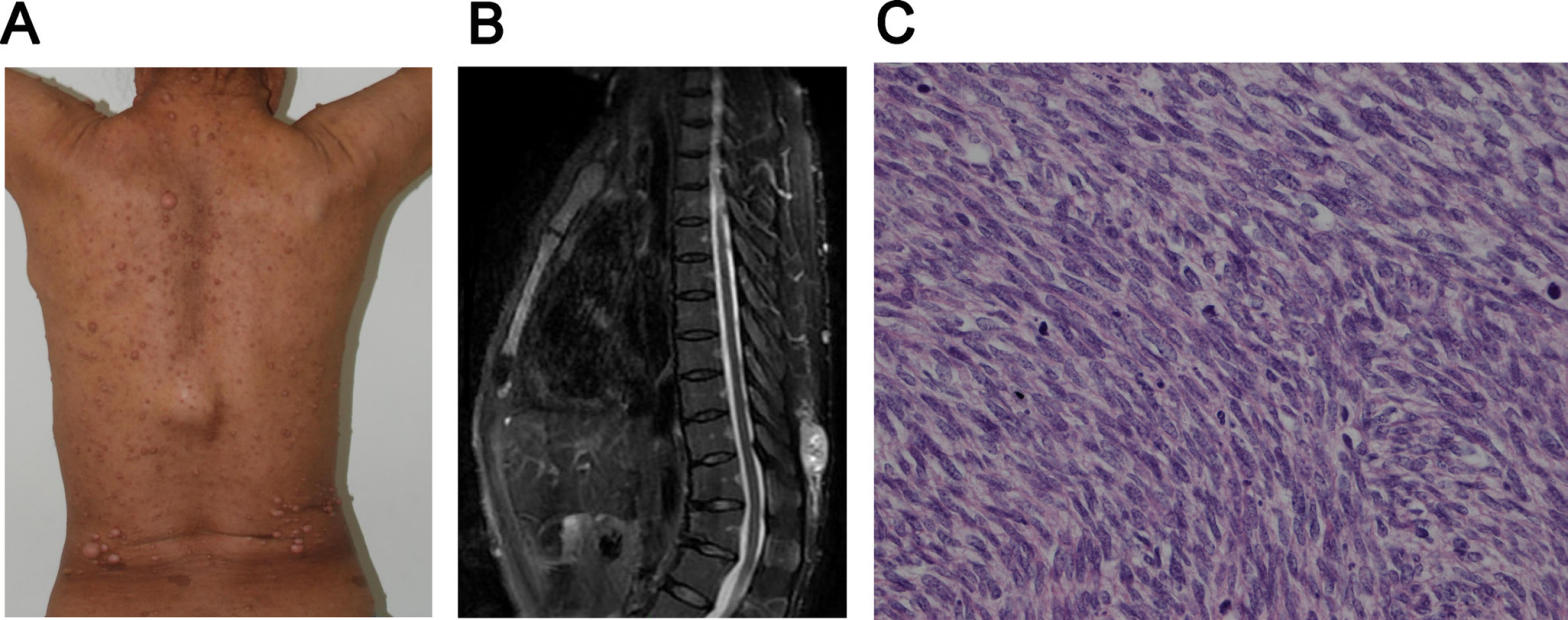
The clinicopathological features of MPNST with NF1

A total of 159 patients with MPNST were enrolled into the study. Patient characteristics were summarized in Table 1. The median age was 40 (range: 5–76) years. The ratio of male to female patients was 1.04:1. In the series, 69 (43.4%) patients presented with primary tumors only, whereas 76 (47.8%) patients were accompanied with recurrent tumors and 14 (8.8%) with distant metastasis. The percentages of NF1 associated MPNST and sporadic MPNST patients were 44.0% and 47.8%, respectively. The remaining 8.2% of enrolled patients were RT-induced. Similar to the foreign current reports, most NF1 patients in the study showed typical clinical and pathological features, such as cutaneous neurofibromas, multiple café-au-lait spots (Figure 1A), a large, lobulated soft tissue mass with heterogeneous signal intensity in axial T2-weighted magnetic resonance image (Figure 1B) and spindle cell morphology with a fascicular pattern (Figure 1C).

**Table 1:** Overall patient, tumor, pathologic characteristics and distribution of events in 159 patients with MPNST

Factor	*n*	% of Total
Presentation status		
Primary	69	43.4
Recurrent	76	47.8
Metastasis	14	8.8
Age-year		
≤ 40	80	50.3
> 40	79	49.7
Gender		
Male	81	50.9
Female	78	49.1
NF1 status		
With NF1	70	44.0
Without NF1	89	56.0
Tumor location		
Head and neck	52	32.7
Trunk	55	34.6
Extremity	52	32.7
Tumor size		
< 5 cm	62	39.0
5–10 cm	64	40.2
> 10 cm	53	20.8
Depth		
Superficial to fascia	81	50.9
Deep to fascia	78	49.1
AJCC stage		
I	35	22.0
II	50	34.6
III	60	37.7
IV	14	8.8
Survival status		
Died of disease	89	56.0
Alive with disease	23	14.5
Alive without disease	47	29.5

Incidence estimates of tumor-free survival and overall survival for all 159 patients									
MPNST	*n*	Tumor-free survival rate				Overall survival rate			
		1-yr	2-yr	3-yr	5-yr	1-yr	2-yr	3-yr	5-yr
Overall	159	-	-	-	-	63.0	56.0	50.0	43.0
Presentation status									
Primary	69	37.0	37.0	37.0	29.0	58.0	50.0	45.0	36.0
Recurrent	76	43.0	40.0	40.0	36.0	72.0	65.0	62.0	54.0
Metastasis	14	-	-	-	-	43.0	36.0	14.0	14.0

Among the 159 patients, 19 individuals received no surgery: 14 stage IV cases of the disease with distant metastasis that could not get any obvious benefit from surgery, and 5 patients received palliative treatment for unsuitable health conditions. 140 patients (88.1%) with localized tumors underwent a complete resection. The decision of receiving radiotherapy and/or systemic chemotherapy was made by surgeons, radiologists, physicians, influenced by willingness of patients themselves. In the study, among the 140 patients, 60.7% underwent 15–76 Gy of radiotherapy to primary and/or recurrent lesions before, intra or after surgery. A total of 39 patients were administrated with regimens of doxorubicin and ifosfamide, with or without dacarbazine. 3 patients were stage I and their prognosis were quite well. 36 patients were stage II or stage III. So we conduct the role of chemotherapy among stage II and stage III cases.

**Figure 2:**
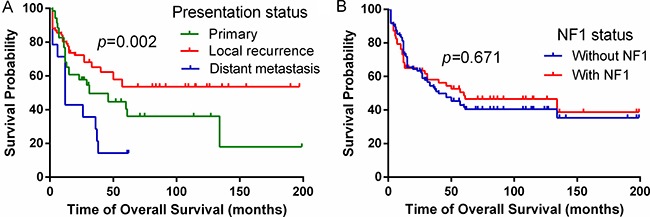
Clinical factors affecting OS in all MPNST patients (**A**) Univariable analysis demonstrated that patients presenting with metastasis harbor the worst prognosis (*p* = 0.002). (**B**) No difference in outcome was observed when comparing NF1 status (*p* = 0.671). Kaplan-Meier curves are depicted.

**Figure 3:**
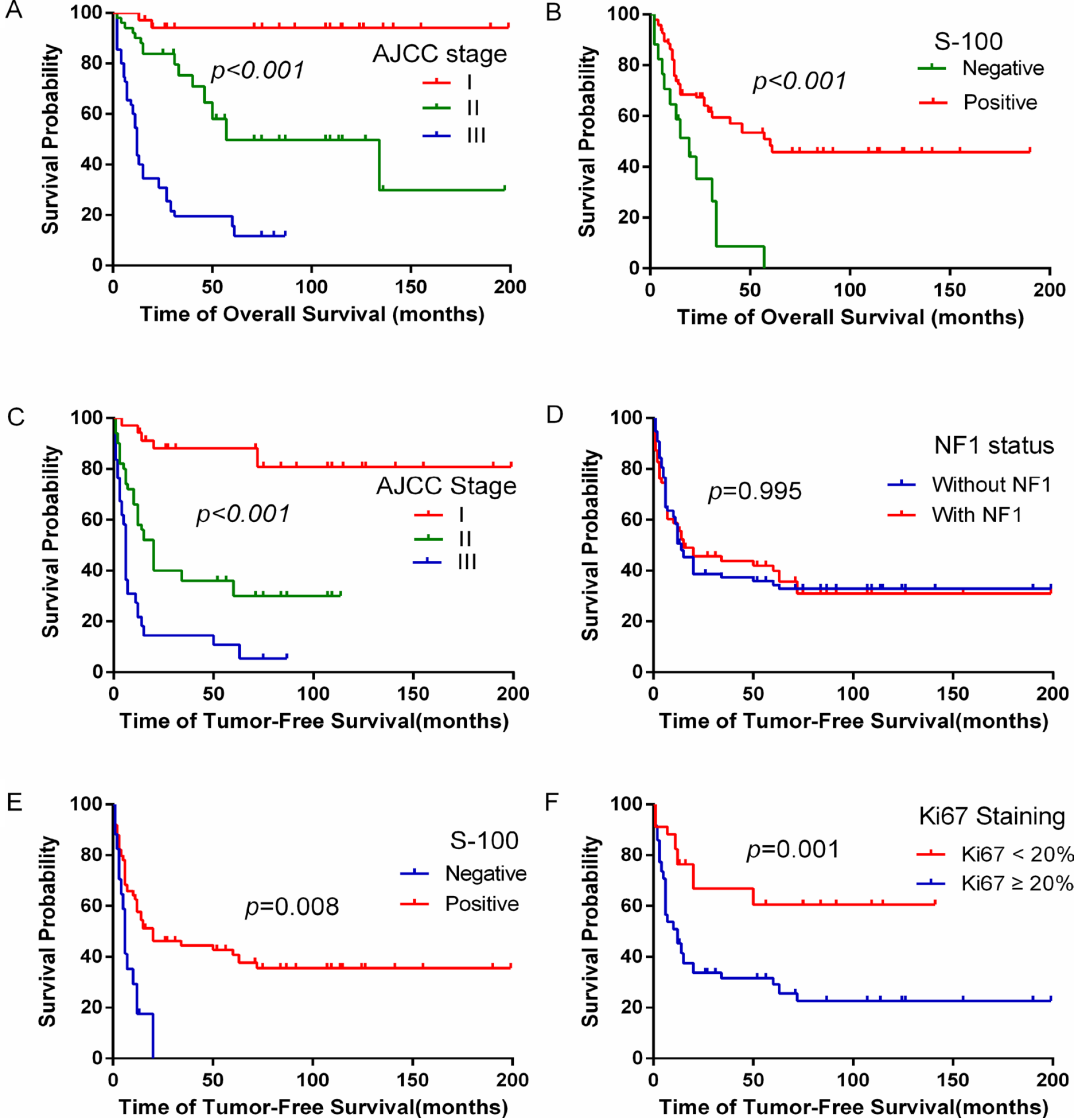
The effect of clinicopathological factors on overall survival and tumor-free survival of MPNST patients (**A**) Patients with the late AJCC stage had worse overall survival rate. (**B**) MPNST patients with S-100 negative had significantly worse overall survival. (**C**) MPNST patients with the late AJCC stage had lower tumor-free survival. (**D**) No difference in tumor-free survival was observed when comparing NF1 statuses. (**E**) MPNST patients with S-100 negative had significantly worse tumor-free survival. (**F**) MPNST patients with Ki67 ≥ 20% had significantly worse tumor-free survival.

The median follow-up time was 31.0 (range: 2.0–199.0) months. The 3- and 5-year overall survival rates of the whole group were 50.0% and 43.0%, respectively (Table 1). 2- and 3-year OS for patients with metastatic disease were 36.0% and 14.0%, which were significantly worse than the localized disease patients (*p* = 0.002, Figure 2A). NF1 associated MNPST did not show survival advantage compared with those without NF1 presentation (*p* = 0.671, Figure 2B and p = 0.995, Figure 3D). Additionally, the TFS and the OS of radiation-induced MPNST compared to the sporadic and NF1 -associated are similar (*p* > 0.05 for both, Table 2).

**Table 2:** Prognostic factors for tumor-free survival and overall survival in 140 completely resected patients with MPNST

Factor	*N*	%	Tumor-free survival			Overall survival		
			HR	95% CI	*P*	HR	95% CI	*P*
Age								
≤ 40	71	51.0						
> 40	69	49.0	1.085	0.708–1.662	0.708	0.954	0.598–1.521	0.842
Gender								
Male	66	47.1						
Female	74	52.9	0.977	0.648–1.473	0.913	1.177	0.747–1.856	0.482
Tumor location								
Head and neck	51	36.4						
Trunk	46	32.9	0.761	0.485–1.193	0.233	0.569	0.338–0.959	0.034^*^
Extremity	43	30.7	0.218	0.120–0.397	< 0.001^*^	0.226	0.120–0.426	< 0.001^*^
Tumor size								
≤ 5 cm	58	41.4						
> 5 cm	82	58.6	2.089	1.337–3.263	< 0.001^*^	1.450	0.904–2.324	0.123
Depth								
Superficial to fascia	67	47.9						
Deep to fascia	73	52.1	4.120	2.596–6.539	< 0.001^*^	3.815	2.293–6.348	< 0.001^*^
NF-1 status								
Without NF-1	77	55.0						
With NF-1	63	45.0	0.995	0.659–1.501	0.980	1.024	0.649–1.616	0.919
AJCC stage								
I	35	25.0						
II	50	35.7	6.036	2.522–14.446	< 0.001^*^	11.134	2.625–47.217	0.001^*^
III	55	39.3	14.398	6.103–33.968	< 0.001^*^	40.509	9.659–169.893	< 0.001^*^
Margin status								
Negative	123	87.9						
Positive	17	12.1	2.327	1.331–4.070	0.003^*^	2.472	1.401–4.362	0.002^*^
Radiation								
Yes	85	60.7						
No	55	39.3	1.453	0.959–2.200	0.078	2.407	1.297–3.233	0.002^*^
Chemotherapy								
Yes	36	34.3						
No	69	65.7	0.995	0.642–1.543	0.984	1.139	0.700–1.848	0.603

MPNST	Incidence Estimates of Tumor-free survival and Overall Survival for 140 Completely Resected Patients								
	*N*	Tumor-free survival rate				Overall survival rate			
		1-yr	2-yr	3-yr	5-yr	1-yr	2-yr	3-yr	5-yr
	140	42.0	40.0	40.0	34.0	65.0	58.0	54.0	45.0
Median Time (months)			18.2				57.0		

### Prognostic factors for overall survival

Till the latest follow-up, 56.0% (89/159) of all patients died of MPNST. Death rate of patients with metastatic disease was much higher than that of patients with localized tumor (85.7% vs. 51.0%, *p* < 0.05). Since patients with metastases presented significantly worse survival outcomes, these 14 individuals were therefore excluded from further analysis. 5 patients who had inadequate organ functions received no surgery, and they were also excluded. The 5-year OS rate of 140 patients who underwent a complete resection was 45.0%.

To identify additional factors impacting the outcome of MPNST, further analysis was conducted among patients who received surgeries. Univariate analysis showed that the location and the depth of tumor, AJCC stage, margin status, S-100 and Ki67 (Figure 3F) staining independently affected OS (*p* < 0.05, Tables 2 and 4). However, by multivariate analysis, only AJCC stage (Figure 3A) and S-100 (Figure 3B) were factors associated with prolonged OS (*p* < 0.05, Table 5).

**Table 3:** Biomarker distribution and statistical significance in NF1 and MPNST tumor tissues

Marker	NF1 (*n* = 50)		MPNST (*n* = 112)		*P*
	Staining Negative *n* (%)	Staining Positive *n* (%)	Staining Negative *n* (%)	Staining Positive *n* (%)	
S-100	1 (2.0)	49 (98.0)	17 (15.2)	95 (84.8)	0.014^*^
Ki-67	29 (96.7)	1 (3.3)	34 (42.0)	47 (58.0)	< 0.001^*^
Vimentin	1 (6.7)	14 (93.3)	3 (4.3)	66 (95.7)	0.702
NF	15 (68.2)	7 (32.8)	12 (52.2)	11 (47.8)	0.273
GFAP	13 (91.9)	8 (8.1)	25 (78.1)	7 (21.9)	0.200

**Table 4:** Univariable cox proportions analysis for markers associated with MPNST tumor-free survival and overall survival for patients with localized tumors

Prognostic Factor	*N*	%	Tumor-free survival			Overall survival		
			HR	95% CI	*P*	HR	95% CI	*P*
S-100								
Positive	95	84.8						
Negative	17	15.2	2.492	1.387–4.479	0.002^*^	2.903	1.572–5.360	0.010^*^
Ki67 Staining								
< 20%	34	42.0						
≥ 20%	47	58.0	2.244	1.153–4.369	0.017^*^	3.494	1.830–6.668	< 0.001^*^
Vimentin								
Positive	62	95.4						
Negative	3	4.6	1.225	0.337–3.268	0.692	1.532	0.343–6.253	0.560
NF								
Positive	11	47.8						
Negative	12	52.2	0.930	0.580–1.520	0.734	1.004	0.336–2.999	0.994
GFAP								
Positive	7	21.8						
Negative	25	78.2	1.287	0.144–11.504	0.638	0.537	0.018–3.203	0.520

**Table 5:** Multivariate analysis of prognostic factors for 140 completely resected patients with MPNST

Prognostic Factor	Tumor-free survival			Overall survival		
	HR	95% CI	*P*	HR	95% CI	*P*
Tumor location						
Head and neck	1.000			1.000		
Trunk	0.752	0.312–1.814	0.526	1.149	0.474–2.787	0.758
Extremity	0.292	0.081–1.046	0.059	0.308	0.065–1.455	0.137
Depth						
Superficial to fascia	1.000			1.000		
Deep to fascia	2.103	0.755–5.861	0.155	3.157	0.949–10.505	0.061
Tumor size						
≤ 5 cm	1.000			-		
> 5 cm	1.525	0.677–3.435	0.308	-	-	-
AJCC stage						
I	1.000			1.000		
II	3.784	1.268–11.290	0.017^*^	2.988	0.590–15.141	0.186
III	16.945	4.680–61.350	< 0.001^*^	35.881	7.135–180.442	< 0.001^*^
S-100						
Negative	1.000			1.000		
Positive	0.236	0.081–0.669	0.008^*^	0.151	0.042–0.542	0.004^*^
Ki67						
< 20%	1.000			1.000		
≥ 20%	3.818	1.722–8.464	0.001^*^	1.885	0.826–4.304	0.132
Margin status						
Negative	1.000			1.000		
Positive	1.696	0.610–4.716	0.311	1.892	0.650–5.504	0.242
Radiation						
Yes	-			1.000		
No	-	-	-	1.561	0.734–3.320	0.247

### Prognostic factors for tumor-free survival

The 3-and 5-year TFS rates for the 140 patients were 40.0% and 34.0%, respectively. Most patients experienced local recurrence or distant metastasis during their follow-up. 42.9% experienced local relapse and the median time to relapse was 6.0 months. The 3-year distant relapse rate was 49.3%, and 47.9% developed distant metastases at a median time of 8.0 months. The most common site of distant metastasis was the lungs, which was documented in 35 patients (25.0%). Other common metastatic sites were the bone, brain, and liver, which occurred in 12.1%, 7.1%, and 5.0% of all patients.

Several possible prognostic factors predicting disease progression by analyses of univariable Cox proportions were found. The extremities and the superficial location of MPNST, their early AJCC stage, margin negative, administration of radiotherapy, S-100 positive and Ki67 < 20% were associated with lower recurrence/metastatic rates and prolonged TFS (Table 2). On multivariate analyses, patients with advanced AJCC stage (Figure 3C) and S-100 positive (Figure 3E) had a higher tendency of developing local recurrence and metastases (Table 5).

### Biomarker expression in patients with MPNST

**Figure 4:**
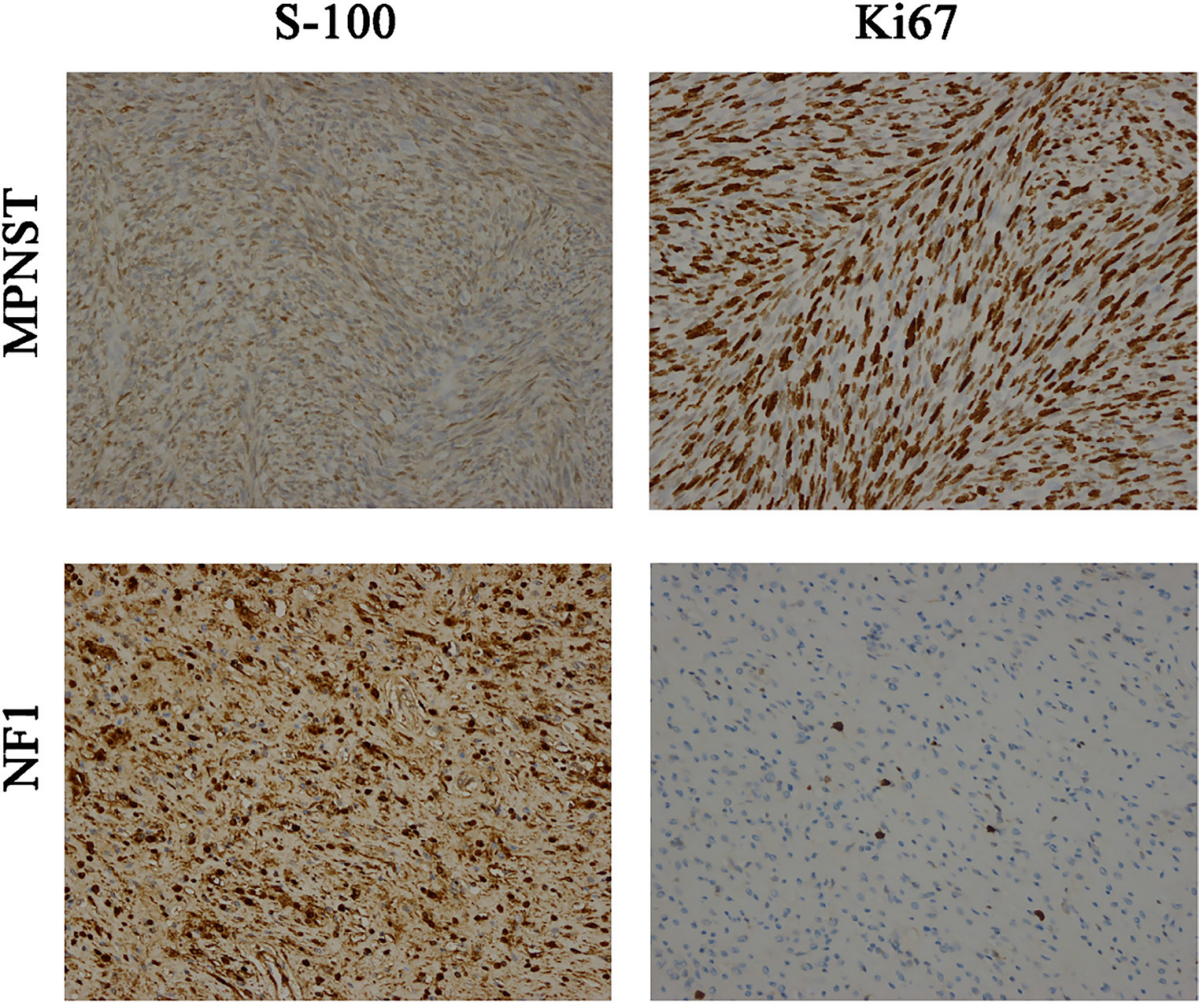
Protein Expression of S-100 and Ki67: in MPNSTs and NF1 (200X), separately

It is important to identify MPNST molecular markers for diagnostic value. To achieve this goal, firstly, histologic specimens of 50 NF1 patients diagnosed in the hospital in the same period were collected. Univariate analysis was performed to evaluate possible correlations between biomarker expression and histologic diagnosis (NF1 vs. MPNST). The percentage of NF1 and MPNST specimens exhibiting biomarker positivity (or in case of Ki67 staining, percentage of specimens exhibiting positive staining in ≥ 10% of tumor cells) was calculated (Figure 4). Positive S-100 expression was demonstrated in 84.8% of MPNST samples compared with 98.0% of neurofibroma specimens (Table 3). In summary, the expression of S-100 and Ki67 staining differentiated MPNST from NF1 at a significance of *p* < 0.05. Though Vimentin, NF and GFAP were slightly higher in positivity in NF1 specimens, they did not reach statistical significance (Table 4).

## DISCUSSION

MPNSTs are rare soft tissue sarcoma. Knowledge of their clinical outcome is limited, which impedes the ability to construct tumor-specific sensitive prognostic paradigms. This is one of the largest studies published within the last 30 years evaluating MPNST populations consisting of at least 100 patients [4, 6, 12, 13]. Data showed the unfavorable outcome of MPNST, as well as the diagnostic value of S-100 and Ki67 in MPNST. Most importantly, it had been the largest retrospective study of Chinese populations to identify clinical and molecular predictors for MPNST to date.

In the study, the 3- and 5-year OS rates were 50.0% and 43.0%, respectively for the whole group, and 54.0% and 45.0%, respectively for patients who received tumor resection. The survival of Chinese patients with MPNST after multidisciplinary treatments was unsatisfactory, which was no difference from the published data with the 5-year survival rates ranging from 35 to 52% [4–6, 12].

The percentages of NF1 associated MPNST and RT-induced were 47.8% and 8.2%, respectively. 44% of patients in the series had NF1. Other studies in the literature reported that it consisted of 22% to 52% of their study samples [4–6, 13]. It is no doubt that patients with NF1 are at increased risk of developing MPNST. There are conflicting reports as to whether malignant MPNST in NF1-patients with have worse prognoses than MPNST in non-NF1 patients. Porter and colleagues found that NF1 (*p = 0*.007) remained independent predictors of poor outcome and they recommended that NF1 be taken into account during MPNST staging [14]. Kolberg conducted a survival meta-analysis for > 1800 MPNST patients with and without NF1 [10]. The compiled literature from 1963 to 2012 indicated a significantly worse outcome of MPNST in patients with NF1 syndrome compared with that in non-NF1 patients. However, survival for the NF1 patients has improved in the last decade, and the survival difference is diminishing. In the study, there was no significant difference between the survival of patients with and without NF1 (*p* > 0.05). Additionally, in our study, it has no significance of the TFS and the OS of radiation-induced MPNST compared to the sporadic and NF1 -associated MPNST. However, LaFemina and colleagues reported on 105 patients with MPNST and found that NF1-associated and sporadic MPNSTs may be associated with improved disease specific survival compared to RT-induced tumors [15].

Noteworthily, the study found several prognostic factors for survival of MPNST patients. Data showed that the location, the depth, the size, the AJCC stage of the tumor, and S-100 were associated with the tumor-free survival. Whereas, late AJCC stage and S-100 negative were independent unfavorable factors affecting OS. Local recurrence or distant metastasis were much common in patients whose tumor was deeply located, especially in the head and neck region, tumor size > 5 cm, advanced AJCC stage, and/or Ki67 ≥ 20%. These results from Chinese patients in the report were in accordance with the reported data worldwide.

Some molecules including S-100, Ki67 may be useful in assessing the prognosis of MPNST. S-100 was considered a marker of neural crest differentiation and widely used to identify nerve sheath tumors from other soft-tissue neoplasms [16]. However, S-100 was negative in some MPNST patients as reported previously [12], which was considered the differentiation of Schwann cells [17]. In the series, 15.2% of the tumors were identified as S-100 negative and 84.8% positive. Further, it was found that S-100 negative was an independent prognosis factor, with a 3.24-fold increased risk of recurrence or metastasis, and a 5.62-fold increased risk of mortality. Ki67 was reported as a marker of cell proliferation, and had been used for predicting the prognosis of some tumors, such as breast cancers and lymphomas. Previous studies also confirmed upregulation of Ki67 in MPNST when compared with benign schwannoma [5]. In the report, 58.0% of MPNST tumors were identified as Ki67 ≥ 10%, compared with 3.3% of NF1. In prognostic analysis, Ki67 ≥ 20% was proven to be an independent prognostic factor, with a 2.81-fold increased risk of mortality, compared with Ki67 < 20% MPNST (*p* = 0.001). The above conclusion was quite similar to the study from Kolarov [18]. It was an agreement that Vimentin was a maker of epithelial-mesenchymal transition. Therefore, it is higher in many malignant tumors, such as prostate cancer, breast cancer and colorectal cancer [19]. In the study, Vimentin, NF, and GFAP did not express significant differences in content between MPNST and NF1. Also, it was confirmed that Vimentin, NF, and GFAP were not prognostic factors for postoperative TFS and OS.

The mainstay of therapy for MPNST is surgical resection with the goal of achieving complete removal with negative margins. Many authors confirmed that total resection with a clear margin could reduce the recurrence rate and improve the prognosis of MPNST [4, 20–22]. In the series, negative margins could improve both the OS (*p* = 0.002) and the TFS (*p* = 0.003) on analyses of univariable Cox proportions (Table 4). But it was not an independent prognostic factor via multivariate analysis (*p* > 0.05, Table 5). Radiation therapy is an important adjunct to surgery in improving local control, and may be administered in the neoadjuvant or adjuvant setting, and as intraoperative therapy in centers with the available resources [23]. Although improvement in rates of local control have been seen with adjuvant radiation therapy, only one study out of Milan, Italy has been able to demonstrate that lack of radiation therapy predicts decreased disease specific survival [4]. In the data, adjuvant radiation therapy (HR: 0.688) could improve the OS rate, but it did not reach statistical significance (*p* = 0.078). The administration of adjuvant radiation therapy was associated with an improved TFS on univariate analyses (*p* = 0.002), though it had no impact on OS on multivariate analyses. The role of chemotherapy remains controversial. Some studies supported the effect of chemotherapy in MPNSTs [12, 24], while others considered it as useless [4, 6]. Generally, first-line chemotherapy regimen was doxorubicin-based [25]. Kroep conducted a research analyzing the response and survival of different chemotherapy regimens in patients with advanced MPNST [26]. They came to the conclusion that the doxorubicin-ifosfamide combination had the best response rate (HR: 6.283, 95% CI: 2.342–16.852). In that study, the administration of chemotherapy in MPNST patients had no impact on the survival. That patient sample group was quite small, though (Table 2).

In summary, despite combined multimodality therapy, MPNST behaves as an aggressive sarcoma with a propensity to recur locally or to metastasize to distant sites. It is believed that better survival outcomes were directly related to the high rate of negative margins combined with some certain clinical characteristics. From data of the hospital, tumor complete resection with MPNST patients is encouraged. The role of multidisciplinary approaches including adjuvant chemotherapy and/or radiotherapy need more randomized clinical studies to confirm.

## MATERIALS AND METHODS

The clinical data of MPNST patients who were pathologically diagnosed and treated at Cancer Institute & Hospital, Chinese Academy of Medical Science from January 1999 to January 2016 was retrospectively reviewed. The study has been approved by the Ethics Committee in our hospital. For a tumor to be considered a NF1-associated MPNST. Firstly, its presence was determined on the basis of established NIH criteria [4], ie, patients with NF1 had at least 2 of the following: ≥ six cafe-au-lait macules (> 5 mm before puberty, > 15 mm after puberty), skin-fold freckles (groin, axilla, base of neck), ≥ 2 neurofibromas (1 plexiform), skeletal dysplasia (orbital or tibial), Lisch nodules (iris hamartomas), optic gliomas, and family history. For a tumor to be considered a sporadic MPNST, it must have met at least one of the following criteria: [5] (1.) originated in a peripheral nerve; (2.) originated in a pre-existing nerve sheath tumor (rare, other than neurofibroma in NF1); (3.) exhibited ultrastructural features of Schwannian differentiation. Multiple systems of histologic grading have been applied to MPNST with variable success. Grading of MPNST has not been shown to be of clinical utility under the French Federation of Cancer Centers (FNCLCC) system [5]. And It is a pity that grading of MPNST is not routinely performed at our hospital. All patients were verified by two different senior pathologists. Patient clinical data included age, sex, tumor location, largest diameter of tumor, clinical AJCC (American Joint Committee on Cancer) stage of tumor, time to recurrence or metastatic status, treatments and outcomes.

### Immunohistochemical methods

The 112 formalin-fixed paraffin-embedded MPNST tissues were sectioned at 4 um and mounted on to charged glass slides for immunohistochemical stainings as previously described with minor modification. The concentrations of the diluted antibodies were 1:75 for S-100 (PL0401286, PLLABS), Ki67 (orb67076, biorbyt), Vimentin (3295s, Cellsignal), neurofilament (NF) (PAB19367, Bio-Swamp), and glial fibrillary acidic protein (GFAP) (orb26155, biorbyt). The protein expressions were estimated by two different senior pathologists.

### Statistics

Data were analyzed using SPSS version 19.0 (SPSS, Inc. Chicago, IL, USA). Tumor-free survival (TFS) was calculated from the date of diagnosis to the date of local recurrence, or distant metastasis, or last follow-up, or date of death from any cause. Overall survival (OS) was calculated from the date of diagnosis to the date of death, or last follow-up, whichever occurred first. TFS and OS probabilities were estimated by the Kaplan-Meier methods. Log-rank tests were used to compare the OS probability and TFS probability between groups. Cox proportional-hazards regression (Cox PH) analysis was performed to calculate the hazard ratio and 95% confidence interval. The univariable Cox PH model were fitted to evaluate the predictive effect of clinical characteristics and biomarkers. The significant predictors in univariable models were candidate variables in the multivariable Cox PH model. The cutoff *p* value was set as 0.05.

## Abbreviations

MPNST: malignant peripheral nerve sheath tumors
OS: overall survival
TFS: tumor-free survival
GFAP: glial fibrillary acidic protein
NF: neurofilament
AJCC: American Joint Committee on Cancer
HR: hazard ratio
CI: confidence interval
